# The Surgical Workforce and Surgical Provider Productivity in Sierra Leone: A Countrywide Inventory

**DOI:** 10.1007/s00268-016-3417-1

**Published:** 2016-01-28

**Authors:** Håkon A. Bolkan, Lars Hagander, Johan von Schreeb, Donald Bash-Taqi, Thaim B. Kamara, Øyvind Salvesen, Arne Wibe

**Affiliations:** Department of Cancer Research and Molecular Medicine, Norwegian University of Science and Technology, Box 8905, 7491 Trondheim, Norway; Paediatric Surgery and Global Paediatrics, Department of Paediatrics, Clinical Sciences Lund, Lund University, Lund, Sweden; Health System and Policy Research Group, Karolinska Institutet, 171 77 Stockholm, Sweden; Ministry of Health and Sanitation, Freetown, Sierra Leone; Department of Surgery, Connaught Hospital and College of Medicine Allied Health Sciences, University of Sierra Leone, Freetown, Sierra Leone

## Abstract

**Background:**

Limited data exist on surgical providers and their scope of practice in low-income countries (LICs). The aim of this study was to assess the distribution and productivity of all surgical providers in an LIC, and to evaluate correlations between the surgical workforce availability, productivity, rates, and volume of surgery at the district and hospital levels.

**Methods:**

Data on surgeries and surgical providers from 56 (93.3 %) out of 60 healthcare facilities providing surgery in Sierra Leone in 2012 were retrieved between January and May 2013 from operation theater logbooks and through interviews with key informants.

**Results:**

The Sierra Leonean surgical workforce consisted of 164 full-time positions, equal to 2.7 surgical providers/100,000 inhabitants. Non-specialists performed 52.8 % of all surgeries. In rural areas, the densities of specialists and physicians were 26.8 and 6.3 times lower, respectively, compared with urban areas. The average individual productivity was 2.8 surgeries per week, and varied considerably between the cadres of surgical providers and locations. When excluding four centers that only performed ophthalmic surgery, there was a positive correlation between a facility’s volume of surgery and the productivity of its surgical providers (*r*_s_ = 0.642, *p* < 0.001).

**Conclusions:**

Less than half of all of the surgery in Sierra Leone is performed by specialists. Surgical providers were significantly more productive in healthcare facilities with higher volumes of surgery. If all surgical providers were as productive as specialists in the private non-profit sector (5.1 procedures/week), the national volume of surgery would increase by 85 %.

## Introduction

To achieve universal health coverage, it is critical to distribute human resources for health to match the population’s needs [1, 2]. In Sub-Saharan Africa, the surgeon density is reported to range from 0.2 to 1.0 per 100,000 people [3], which is between 15 and 450 times lower than in OECD countries [4]. The lack of skilled providers is considered a main barrier to the expansion of surgical care [5, 6], and there is a particular mismatch between the large unmet need for surgery and the shortage of surgical providers in rural areas in low-income countries (LICs) [7, 8].

To cover the surgical need in LICs only through fully certified surgeons is considered inconceivable in the foreseeable future [9]. An increasingly applied and acknowledged strategy has been to share surgical tasks with other categories of health care workers [9, 10]. In LICs, a wide range of health care workers perform surgical procedures, including surgeons, obstetricians, non-specialist physicians, midwifes, nurses, and associate clinicians [11, 12]. Comprehensive data that on a national scale describe the full scope of such surgical providers in the setting of LICs are lacking [3].

In order to develop policies to make surgery more available in areas where needs are high, data on the domestic distribution and scope of practice of the surgical workforce are needed. The purpose of this study was to identify all surgical providers in an entire LIC, to map the distribution and productivity of this surgical workforce, and to evaluate correlations between the surgical workforce density, productivity, and rates of surgery.

## Methods

This countrywide facility-based study included data on all surgical providers in Sierra Leone in 2012. The Sierra Leone Ministry of Health and Sanitation and the non-governmental organization CapaCare jointly collected the data in collaboration with the Norwegian University of Technology and Science (NTNU). Eligible for inclusion in the study were all health care facilities that performed one or more of the 21 surgical procedures defined in the World Health Organization (WHO) health facility assessment tool SARA [13]. Twelve 4th- and 5th-year medical candidates from the University of Sierra Leone and NTNU interviewed between January 14 and May 20, 2013 all of the facility directors regarding the workforce availability and collected data on the surgeries performed. Procedure-related surgical data were obtained from operation theater logbooks, maternity books, and anesthesia logbooks. The methodology and definitions of categories for the owner and administrative levels of the facilities have been described previously [14].

Of the 60 facilities identified to have performed surgical procedures in Sierra Leone in 2012, surgical records were available in 58 institutions, of which 56 (93.3 %, 56/60) also shared data on the surgical providers [14]. Of the 24,152 surgeries identified, information on the surgical provider category was available for 23,693 (98.1 %) surgeries.

### Definitions

A *surgical procedure* was defined as any procedure requiring general, regional, or local anesthesia performed within an operation theater [15]. A *surgical provider* was defined as a person who, according to the log book, was the principal operator in the included facilities in 2012. A *specialist* was defined as a senior physician who had completed specialist training in surgery, orthopedics, gynecology/obstetrics, ophthalmology, or otolaryngology. A *physician* was defined as a non-specialist holder of a medical degree and included house officers, who are physicians in the 2-year obligatory postgraduation internship. A medical practitioner licensed by the Sierra Leone Nurses & Midwives Board was listed as a *nurse*. All others were defined as *associate clinicians*. A surgical provider with a Sierra Leonean passport was defined as a *domestic provider*; all others were defined as *foreigners*. The term *surgical workforce* refers to all surgical providers.

Surgical providers were quantified as equivalent *full*-*time positions*. A half-time position for 1 year and a full-time position (FTP) for 6 months both counted as a 0.5 FTP. *Productivity* was defined as the weekly number of surgical procedures performed per FTP. Villages with <50,000 inhabitants were considered rural [16]. The neighboring districts of Western Area Urban and Western Area Rural were merged into one district labeled Western Area because of difficulties in distinguishing between the two. The two facilities without surgical human resource data were both located in the country’s smallest district, Bonthe; this resulted in the inclusion of 12 districts in the analysis.

### Analysis

The 2012 projections from the most recent census were used to calculate the density of the workforce [17]. SPSS version 21 and R version 2.13.1 were used for descriptive statistics and statistical analysis. The Chi-square test for trends was used to assess if preference for working in rural areas decreased with higher degree of specialization. Spearman's rank correlation (*r*_s_) was used to explore the relationship between the rate of surgery, the density, and the productivity of surgical providers. The Sierra Leone Ethics and Scientific Review Committee and the Regional Committee for Medical and Health Research Ethics in central Norway (No: 2012/2187) granted ethical clearance for this study.

## Results

### Distribution and density

The Sierra Leonean surgical workforce consisted of 164 FTPs, of which 35.6 % were specialists, 52.3 % were physicians, 3.8 % were nurses, and 8.4 % were associate clinicians. Less than one-quarter (24.8 %) of the total surgical workforce and one-tenth (9.1 %) of the specialists worked in rural areas. More than three-quarters (76.1 %) of the surgical providers were Sierra Leonean nationals. The private non-profit sector employed 43.7 % of the surgical workforce and performed 53.8 % of the surgeries (Table 1).

**Table 1:** Distribution of surgical providers by full-time positions (FTP) in Sierra Leone in 2012

Variable	No. (%)^a^				
	All	Specialist	Physician	Nurse	Associate clinician
	164.4 (35.6)	86.0 (52.3)	6.2 (3.8)	13.8 (8.4)	
Organizational level					
Clinic	17.9 (10.9)	9.4 (16.1)	3.3 (3.9)	2.1 (35.1)	3 (21.8)
District hospital	75.7 (46.0)	19.2 (32.9)	43.7 (50.8)	2 (32.4)	10.7 (78.2)
Referral hospital	70.8 (43.1)	29.8 (51.0)	39 (45.3)	2 (32.4)	0 (–)
Owner					
Governmental	76.3 (46.4)	21.3 (36.3)	50.1 (58.2)	2 (32.4)	3 (21.8)
Private non-profit	71.8 (43.7)	26.4 (45.2)	32.6 (37.9)	4.1 (66.2)	8.8 (63.6)
Private for-profit	16.2 (9.9)	10.8 (18.5)	3.3 (3.9)	0.1 (1.4)	2 (14.5)
Urban/rural					
Urban	123.6 (75.2)	53.2 (90.9)	60.6 (70.4)	4.1 (66.2)	5.8 (41.8)
Rural	40.8 (24.8)	5.3 (9.1)	25.4 (29.6)	2.1 (33.8)	8 (58.2)
District^b^					
Bo	11 (6.7)	6.1 (10.4)	3.3 (3.9)	0.1 (1.4)	1.5 (10.9)
Bombali	12.4 (7.5)	2.4 (4.1)	6 (7.0)	1 (16.2)	3 (21.8)
Kailahun	6 (3.6)	0 (–)	4 (4.7)	1 (16.2)	1 (7.3)
Kambia	3.1 (1.9)	0 (–)	2.1 (2.4)	0 (–)	1 (7.3)
Kenema	7.4 (4.5)	2.3 (4.0)	5 (5.8)	0.1 (1.4)	0 (–)
Koinadugu	1.2 (0.7)	0.2 (0.3)	1 (1.2)	0 (–)	0 (–)
Kono	3 (1.8)	0 (–)	3 (3.6)	0 (–)	0 (–)
Moyamba	2 (1.2)	0 (–)	2 (2.3)	0 (–)	0 (–)
Port Loko	10.4 (6.3)	1.3 (2.3)	7.1 (8.2)	0 (–)	2 (14.5)
Pujehun	5 (3.0)	1 (1.7)	4 (4.7)	0 (–)	0 (–)
Tonkolili	7.6 (4.6)	0.3 (0.6)	3.3 (3.8)	0 (–)	4 (29.1)
Western Area^c^	95.3 (58.0)	44.8 (76.6)	45.3 (52.6)	4 (64.8)	1.25 (9.1)
Nationality					
Domestic	125.2 (76.1)	39.3 (67.2)	66 (76.7)	6.2 (100)	13.8 (100)
Foreign	39.2 (23.8)	19.2 (32.8)	20 (23.3)	0 (–)	0 (–)

^a^Because of rounding, percentages may not total 100

^b^Excluding Bonthe district

^c^Western Area Urban and Western Area Rural combined

**Fig. 1:**
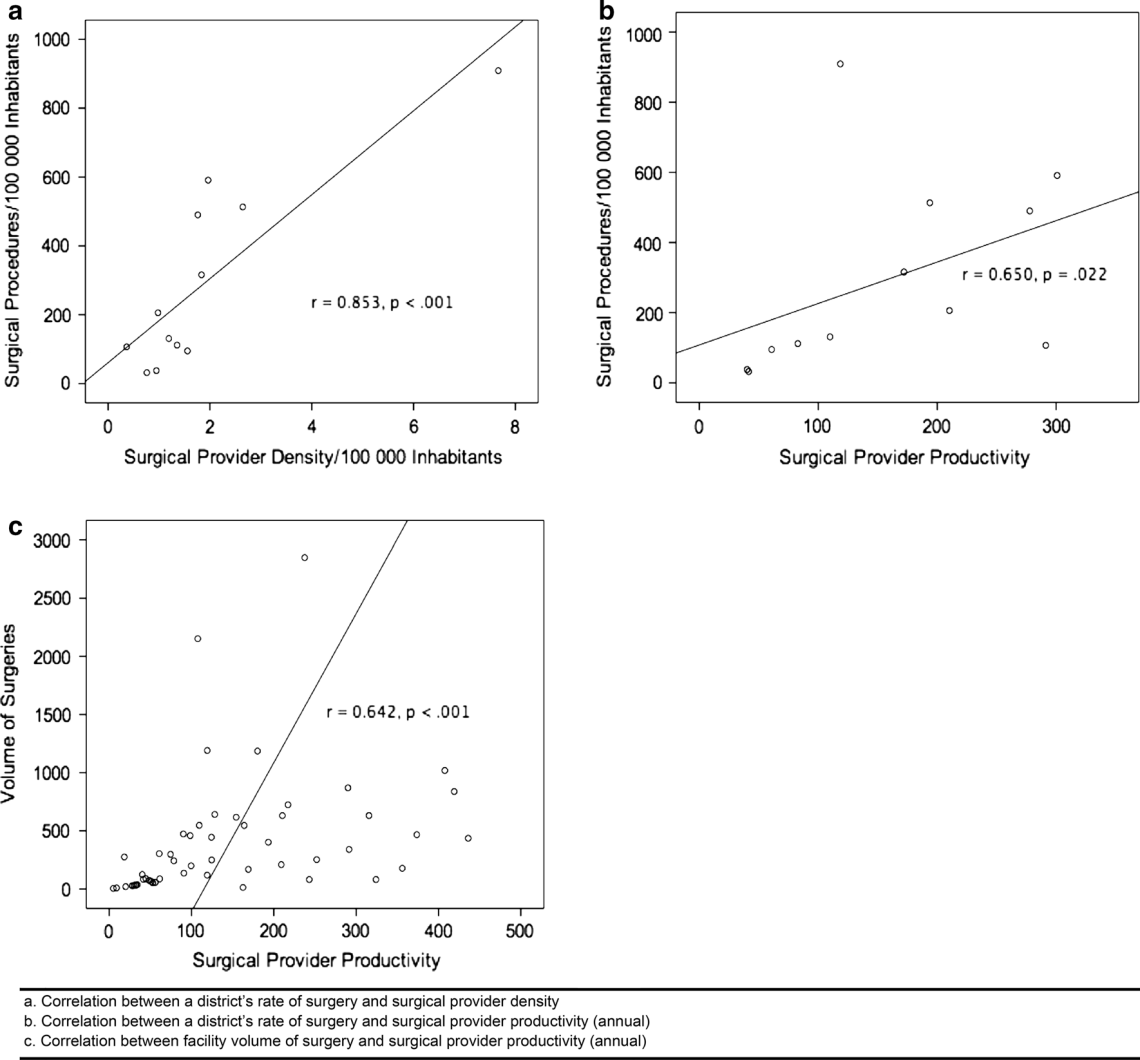
**a** Correlation between a district’s rate of surgery and surgical provider density. **b** Correlation between a district’s rate of surgery and surgical provider productivity. **c** Correlation between a facility’s volume of surgery and its surgical provider productivity

Four of the 12 districts, accounting for more than 1.3 million people (22.1 % of the population), lacked a specialist surgical provider. Six of the districts, accounting for more than 2 million people (34.0 % of the population), had less than one full-time specialist position (Table 1). The national mean density of surgical providers was equivalent to 2.72 FTP per 100,000 inhabitants, of which there were 0.97 specialists, 1.42 physicians, 0.10 nurses, and 0.23 associate clinicians. The overall density of surgical providers was 8.0 times higher in urban areas than in rural areas. The densities of specialists and physicians were 26.8 and 6.3 times higher in urban compared with rural areas (Table 2). The more specialized the provider, the more likely they were to work in urban areas (*p* < 0.001). There was a positive correlation between the rate of surgery and the density of surgical providers at the district level (*r*_s_ = 0.853, *p* < 0.001) (Fig. 1a).

**Table 2:** National, urban, and rural density of surgical providers by cadre and nationality

	Density		
	National	Urban	Rural
Cadres			
Specialist	0.97	3.21	0.12
Physician	1.42	3.65	0.58
Nurse	0.10	0.25	0.05
Associate clinician	0.23	0.35	0.18
Nationality			
Domestic	2.07	5.78	0.68
Foreign	0.65	1.68	0.26
All surgical providers	2.72	7.45	0.93

Density, number of surgical providers per 100,000 inhabitants

### Scope of practice

Specialists performed 47.2 % of all surgeries, while physicians performed 39.4 %, nurses 6.6 %, and associate clinicians 6.8 %. Specialists had the most diverse surgical activity and performed a wider scope of procedures compared with less-specialized cadres. Fifty-three percent of the procedures performed by physicians and 62 % of the procedures performed by the associate clinicians were either a hernia repair or a cesarean delivery. Specialists and physicians performed 95.5 % of orthopedic surgeries. Forty-six percent of all surgical procedures performed by nurses were ophthalmic procedures, mostly cataract surgery (Table 3).

**Table 3:** Surgical procedures by surgical providers

Procedure	No. (%)^a^				
	All	Specialist	Physician	Nurse	Associate clinician
	23,693	11,172 (47.2)	9328 (39.8)	1574 (6.6)	1619 (6.8)
General surgery					
Hernia repair	5127	1709 (33.3)	2511 (49.0)	240 (4.7)	667 (13.0)
Appendectomy	1599	460 (28.8)	1105 (69.1)	7 (0.4)	27 (1.7)
Laparotomy	1009	439 (43.5)	477 (47.3)	23 (2.3)	70 (6.9)
General surgery other	2329	1327 (57.0)	736 (31.6)	87 (3.7)	179 (7.7)
Malignancy surgical	173	156 (90.2)	15 (8.7)	0 (–)	2 (1.2)
Obstetric and gynecology					
Cesarean delivery	4737	1822 (38.5)	2458 (51.9)	118 (2.5)	339 (7.2)
Obgyn other	743	311 (41.9)	413 (55.6)	6 (0.8)	13 (1.7)
Dilatation and curettage	606	293 (48.3)	240 (39.6)	5 (0.8)	68 (11.2)
Hysterectomy	474	217 (45.8)	216 (45.6)	6 (1.3)	35 (7.4)
Incision and drainage abscess	370	168 (45.4)	124 (33.5)	6 (1.6)	72 (19.5)
Obstetric fistula repair	264	171 (64.8)	93 (35.2)	0 (–)	0 (–)
Salpingectomy ectopic pregnancy	172	93 (54.1)	74 (43.0)	1 (0.6)	4 (2.3)
Manual placenta removal	121	21 (17.4)	81 (66.9)	0 (–)	19 (15.7)
Orthopedic surgery					
Orthopedic surgery other	1169	752 (64.3)	334 (28.6)	8 (0.7)	75 (6.4)
Fracture operative	897	725 (80.8)	170 (19.0)	0 (–)	2 (0.2)
Amputation lower limb	203	115 (56.7)	68 (33.5)	1 (0.5)	19 (9.4)
Fracture conservative	154	82 (53.2)	69 (44.8)	0 (–)	3 (1.9)
Ophthalmic surgery	2304	1580 (68.6)	0 (–)	724 (31.4)	0 (–)
Other^b^	306	211 (69.0)	70 (22.9)	7 (2.3)	18 (5.9)
Unknown procedure	936	521 (55.6)	74 (7.9)	335 (35.8)	7 (0.7)

^a^Because of rounding, percentages may not total 100

^b^Procedures performed less than 100/year

### Productivity

Overall, the average productivity was 2.8 surgeries per week. By cadre, nurses were the most productive, performing 4.9 procedures per week, compared with physicians, who performed 2.1 procedures per week. Specialists working in the private non-profit sector performed a mean number of 5.1 surgeries per week, almost twice that of specialists in the governmental sector (2.8/week). For physicians, the productivity was the same in the private non-profit sector and the governmental sector (2.1 and 2.2/week). Specialists performed 2.9 procedures per week in the referral hospitals, almost twice the productivity of the physicians (1.5/week) in the same hospitals. Physicians performed 60 % of all of their surgeries in the district hospitals, with a mean productivity of 2.7 surgeries per week (Table 4).

**Table 4:** Annual volume of surgical procedures and productivity by organizational level and owner

Variable	No. (P)				
	All	Specialist	Physician	Nurse	Associate clinician
Organizational level					
Clinic	4012 (4.3)	2852 (5.8)	198 (1.1)	669 (5.9)	293 (1.9)
District hospital	11,743 (3.0)	3796 (3.8)	6123 (2.7)	498 (4.8)	1326 (2.4)
Referral hospital	7938 (2.2)	4524 (2.9)	3007 (1.5)	407 (3.9)	0 (–)
Owner					
Governmental	9408 (2.4)	3091 (2.8)	5700 (2.2)	406 (3.9)	211 (1.4)
Private non-profit	12,749 (3.4)	6999 (5.1)	3481 (2.1)	1148 (5.4)	1121 (2.5)
Private for-profit	1536 (1.8)	1082 (1.9)	147 (0.8)	20 (4.6)	287 (2.8)
Total	23,693 (2.8)	11,172 (3.7)	9328 (2.1)	1574 (4.9)	1619 (2.3)

*P* productivity, weekly number of surgical procedures per full-time position

If the productivity of all surgical providers could be increased to the same level as that of the specialists in the private non-profit sector, the national volume of surgery in Sierra Leone would increase from 23,693 to ~43,500 (5.1 weekly surgeries × 52 weeks × 164 FTP), an increase of 85 %.

Districts with higher surgical rates had a significantly higher productivity per surgical provider (*r*_s_ = 0.650, *p* = 0.022) (Fig. 1b) [14]. When excluding the four centers that only performed ophthalmic surgery, there was also a positive correlation between a facility’s volume of surgery and the productivity of its surgical providers (*r*_s_ = 0.642, *p* < 0.001) (Fig. 1c).

## Discussion

The density of specialist surgical providers was very low, and within the range of other Sub-Saharan countries [18]. The current specialist surgical workforce represents less than 5 % of the targeted 20 surgical, anesthetic, and obstetric providers the Lancet Commission recommends per 100,000 population [11]. The urban to rural maldistribution of surgical providers was striking, and the imbalance increased with higher degree of medical specialization. As in other resource-poor settings of the world where fully trained surgeons are absent, the patients in Sierra Leone are either cared for by other surgical providers, or they are not cared for at all [3]. From a human resource perspective, strategies to increase surgical capacity include training new surgical specialist providers, sharing surgical tasks with more healthcare workers, and using the entire workforce more efficiently. Our findings indicate that all three approaches are relevant in the Sierra Leonean context.

### New surgical providers

Accredited postgraduate training by the West African College of Surgeons is not presently available in Sierra Leone [19]. Affording domestic physicians this opportunity is crucial, but most likely, it will primarily strengthen the surgical workforce in urban areas, at least initially. Surgical training of non-specialist physicians for the district hospitals remains essential, and surgical specialists play an important role as trainers in this process. It is therefore a concern that specialists perform the majority of their surgeries in the private non-profit sector that traditionally has prioritized service delivery [20]. Physicians perform as few as 1.5 surgeries per FTP per week in the main training hospitals, which are the referral hospitals. This indicates that the referral hospitals, that also provide postgraduate training of house officers might be better utilized as capacity builders for surgery in Sierra Leone. Training specialist surgeons and non-specialist physicians is limited by the country’s low production and retention of medical doctors. As of 2013, a total of 257 had graduated since the establishment of the medical school in 1988, and a substantial proportion is practicing outside of Sierra Leone [21].

### Increasing productivity

Districts with lower volumes of surgery not only had a lower workforce density, but also a less productive surgical workforce, which makes them even more marginalized in terms of surgical output. Surgical productivity also varied considerably between categories of health care workers and work locations, and there might be a potential to utilize the surgical workforce more efficiently. If the productivity of all surgical providers could be increased to the same level as that of the specialists in the private non-profit sector, the national volume of surgery in Sierra Leone would be almost doubled.

The positive correlation between volume of surgery at the district and facility levels and productivity per surgical provider may have several explanations, like quality of surgery, confidence among surgical providers to manage surgical cases, an acceptable infrastructure, and trust between patients and practitioners.

### Task sharing

Due to the towering shortages of surgical providers in the rural areas of Sierra Leone, it is not sufficient to increase the productivity of the few working in this environment. Several findings in the present study support regulated delegation of surgical tasks to less-specialized health care workers. Firstly, surgery is already widely performed by non-specialist physicians, nurses, and associate clinicians. Secondly, all of the nurses and associate clinicians performing surgery are Sierra Leonean health care workers, and nearly all of the procedures performed by associate clinicians are performed at district hospitals, where the surgical need is highest. The retention of associate clinicians at district hospitals is described to be far better than for physicians [22]. Thirdly, hernia repair and cesarean delivery comprise more than 50 % of the surgical volume of physicians and 30 % for specialist providers, and sharing these standardized, high-volume procedures with less-trained providers would allow highly skilled surgical specialists to use their expertise more productively.

The density of specialist surgeons, anesthesiologists, and obstetricians is proposed as an indicator of the surgical workforce [11]. As the present study found that specialists performed fewer than half of the surgeries, it should be noted that this human resource metric underestimates the full range of surgical providers in a low-income country such as Sierra Leone. Data on the subnational distribution of all cadres performing surgeries are required for a more comprehensive understanding of the national response to specialist shortages. Obviously, many other elements in a surgical health care system need to be addressed in order to increase surgical output, such as infrastructure, anesthesia care, supply chain management, affordability, and timely access for patients [11].

### Limitations

This is a comprehensive nationwide inventory of the surgical workforce, but certain limitations exist that are related to the data collection, the definitions, and the volume of surgery. These limitations include the retrospective design, challenges in categorizing some of the health care facilities, and the fact that district rates of surgery were based on the notion that patients have their operation performed in the same district as they reside [14]. The population projection for 2012 is based on the most recent census, performed several years earlier, and represents a potential source of error. Applying FTP instead of headcounts made it possible to adjust for multiple work locations and high turnover rates, and it allowed us to calculate productivity, since the time dimension was included. There could be a potential for the facility directors to overestimate the size of the surgical provider positions, but our findings are consistent with headcounts from 10 governmental hospitals in Sierra Leone in 2012 [19].

When calculating productivity, all surgical procedures were weighted equally. Thus, it is not unexpected that nurses, who mostly performed cataract surgeries, were the most productive providers due to the nature of the surgery performed. The same will probably apply when comparing productivity between administrative levels, as the referral hospitals performed more complex and resource-intensive procedures compared with clinics and district hospitals. This effect is likely less relevant for productivity analysis related to the district level, since clinics with high productivity balance the referral hospital with lower productivity in the same larger urban areas.

## Conclusion

The findings of this study can guide strategies to increase the capacity of the surgical workforce in an LIC. Postgraduate surgical training of specialists is crucial for developing professional champions. There seems to be a potential to improve the exposure and informal training of non-specialist physicians in the referral hospitals. The large untapped potential of using the existing surgical workforce more efficiently should be explored. Expanding the surgical workforce by regulated task sharing to associate clinicians and nurses will promote equity and may match population needs by increasing the surgical workforce where the needs are highest [14]. In short-term workforce planning, it is recommended to engage the private non-profit sector, where specialists currently perform the largest volume of surgeries.

## Appendix

### Box/sidebar: surgery in Sierra Leone

#### Setting

- 14 districts, 6 million inhabitants, 60 % living in rural areas [17].
- Life expectancy is 45.6 years, infant mortality is 117/1,000 live births, and maternal mortality is 890/100,000 live births [23].
- Expenditure on health per capita is US $96 (2012) [24].
- 1054 primary health care units and 51 hospitals [24].

#### Surgical need

- 25 % of the population has a surgical condition requiring medical attention [25].
- 25 % of recent deaths could have been averted by timely surgical care [25].
- More than 90 % of the surgical need is unmet [14].
- There is a 30-fold difference in surgical production between the highest- and lowest-served districts [14].

#### Surgical infrastructure and production in 2012

- Ten consultant surgical providers, median age 60.5 years, were identified in 10 governmental hospitals; all worked in the capital [19].
- 60 facilities offer major surgery [14].
  - 34 district hospitals performed 50 % of the surgical procedures [14].
  - Seven referral hospitals performed 33 % of the surgical procedures [14].
- 54 % of all surgeries were performed by private non-profit actors [14].
